# Supplementary material for: AffyMAPSDetector: a software tool to characterize Affymetrix GeneChip™ expression arrays with respect to SNPs
Source: BMC Bioinformatics. 2007 Jul 30;8:276. doi: 10.1186/1471-2105-8-276 (PMC1959249; doi:10.1186/1471-2105-8-276)
Supplement: Additional file 13 — AffyMAPSDetector v1 source code. AffyMAPSDetector source code. [file 1471-2105-8-276-S13.zip › AffyMAPSDetector_v1_src/doc/affychip/snpdetector/model/package-use.html]

Uses of Package affychip.snpdetector.model


|  |  |  |  |
| --- | --- | --- | --- |
| **Overview** \| **Package** \| Class \| **Use** \| **Tree** \| **Deprecated** \| **Index** \| **Help** | | |  |
| PREV   NEXT | **FRAMES**    **NO FRAMES**     **All Classes** |  |  |


---


## **Uses of Package affychip.snpdetector.model**

|  |  |
| --- | --- |
| Packages that use affychip.snpdetector.model | |
| **affychip.snpdetector.model** |  |

Classes in affychip.snpdetector.model used by affychip.snpdetector.model

****DataState****
  
          Title: Probe SNP Detector

****GeneInfo****
  
          Title: Probe SNP Detector

****Probe****
  
          Title: Probe SNP Detector

****SnpDetector****
  
          Title: Probe SNP Detector

****SnpInfo****
  
          Title: Probe SNP Detector

---


|  |  |  |  |
| --- | --- | --- | --- |
| **Overview** \| **Package** \| Class \| **Use** \| **Tree** \| **Deprecated** \| **Index** \| **Help** | | |  |
| PREV   NEXT | **FRAMES**    **NO FRAMES**     **All Classes** |  |  |


---
